# Minimal Inter-Fractional Fiducial Migration during Image-Guided Lung Stereotactic Body Radiotherapy Using SuperLock Nitinol Coil Fiducial Markers

**DOI:** 10.1371/journal.pone.0131945

**Published:** 2015-07-09

**Authors:** Yi Rong, Jose G. Bazan, Ashley Sekhon, Karl Haglund, Meng Xu-Welliver, Terence Williams

**Affiliations:** 1 Department of Radiation Oncology, University of California Davis Comprehensive Cancer Center, Sacramento, CA, 95817, United States of America; 2 Department of Radiation Oncology, The Ohio State University Wexner Medical Center, Columbus, OH, 43210, United States of America; Taipei Medical University, TAIWAN

## Abstract

**Objectives:**

Stereotactic body radiotherapy (SBRT) is being increasingly used for the treatment of patients with lung cancer or lung metastasis who are medically unfit to undergo resection. In order to improve accuracy and confidence in targeting tumors, many centers rely on fiducial implantation. We evaluated the migration of a novel fiducial marker specifically designed for lung tissue implanted via electromagnetic navigation bronchoscopy (ENB).

**Methods:**

We retrospectively quantified the individual and group migrations of SuperLock nitinol coil fiducials for 15 patients receiving lung stereotactic body radiotherapy (SBRT), in order to evaluate the reliability of using these fiducials as a target surrogate for cases where tumors cannot be clearly delineated on cone beam CTs (CBCTs). For each fraction, we compared the individual and group migrations of the fiducials between the planning CT and the acquired CBCT. The group migration was defined as the distance between the centroids of the fiducial group and GTV.

**Results:**

A total of 16 lung targets were included in our study for these 15 patients (one patient with two targets). Of 55 fiducials placed, we observed a 100% retention rate. The mean individual migration was 1.87 mm (range, 0.63–5.25 mm) with a standard deviation of 1.26 mm. The mean group migration was 1.94 mm (range, 0.03–6.19 mm) with a standard deviation of 1.45 mm. Overall, there was minimal change in the relative locations of the markers with respect to each other, as well as to the target.

**Conclusions:**

We found that the SuperLock nitinol coil fiducial marker positions are stable throughout the radiation treatment, and can be used as a reliable surrogate to target, and to avoid geometric misses during gated treatments.

## Introduction

In the modern era of image guided radiotherapy (IGRT), techniques that seek to further refine the accuracy of treatment delivery have become a highly researched topic. Advancements in areas such as patient immobilization, image acquisition, as well as target localization have contributed to a substantial reduction in both inter- and intrafraction variation, allowing for more consistent and accurate treatment [1,2]. One of the most widely investigated applications of IGRT involves the management of respiration-induced organ motion. Early studies examining lung and liver movement during the respiratory cycle through the use of implanted fiducial surrogate-based real-time tracking, fluoroscopy, and 4D computed tomography (4DCT) demonstrated organ motion of up to 2 to 3 cm, particularly in the cranial-caudal dimension [3–9]. Similar motion has been observed for upper abdominal organs. For example, pancreas motion up to 2–4 cm has been reported based on ultrasound measurements [10].

Respiratory motion affects most thoracic and abdominal tumor sites when treating with radiation, including lung cancer. In the United States alone, primary lung malignancies constitute 50–60% of most new invasive cancers [11]. Non-small cell lung cancers (NSCLC) account for 80–85% of these cases, with 10–15% falling into the category of early-stage, localized disease. For patients diagnosed with early stage, medically-inoperable NSCLC, stereotactic body radiotherapy (SBRT) has brought promising results, yielding low local recurrence rates ranging from 3% to 20% [12–16]. SBRT, also known as stereotactic ablative radiation therapy (SABR), delivers a high dose per fraction to an extra-cranial target in a small number of fractions [17]. In addition to the high dose ablating the tumor cells, recent advances in radiation therapy physics enable a steep dose gradient outside the tumor for limited normal tissue damage, thus optimizing the therapeutic ratio [13].

Given widespread acceptance of SBRT in the management of early-stage inoperable NSCLC (as well as the increasingly common use of SBRT in oligometastatic disease), there has been an increasing focus on technologies that allow for tumor tracking with respiratory motion, in order to further reduce the normal tissue damage by minimizing treatment margins required for a moving target [1–3]. During an SBRT treatment, a challenge is the absence of high radiopaque contrast in 2D radiographic imaging for pulmonary lesions [18]. It has been reported that it is difficult to visualize lung tumors smaller than 2.4 cm using 2D radiographic or fluoroscopic images [19]. Such situations may benefit from bronchoscopically-implanted fiducial markers near the tumor to improve tumor localization. The usefulness of such markers, however, can be negated by their potential migration [18,20,21]. The potential for fiducial migration over the course of treatment is a recognized drawback to this method that could result in inaccurate dosing to the tumor and surrounding tissues. The SuperLock (Covidien, Inc., Minneapolis, MN, USA) gold fiducial marker was specifically designed with a nitinol wire to minimize migration in the lung tissue. The primary objective of the present study is to retrospectively quantify the individual and group migration of SuperLock nitinol coil fiducial markers for patients receiving lung SBRT, thereby evaluating the reliability of using these fiducials as a target surrogate in specific cases where tumors cannot be clearly delineated on cone beam CT (CBCT).

## Material and Methods

### Ethics statement

The study was approved by the Ohio State University Cancer Institutional Review Board (IRB, 2014C0070). Patient records were anonymized prior to analysis. Patient consent was not required since this was a retrospective study with full waiver.

### Patient characteristics

A total of fifteen consecutive patients treated with gated SBRT between 2012 and 2014 with primary NSCLC and lung oligometastases were included in the study. The 15 patients underwent 15 simulation CTs and 58 CBCTs. All of these patients received SBRT with breath-hold gated treatment for a total of 45–55 Gy in 3–5 fractions. Patient demographic information is listed in Table 1. Patient age at treatment initiation ranged from 49 to 82 years old. Tumor size ranged from 0.8 to 3.6 cm in diameter, based on the most recent CT or PET/CT prior to treatment. Patient #7 had two lesions, one in the left lung, and another in the right lung. Therefore, a total of 16 target lesions were studied and analyzed.

**Table 1:** Patients’ characteristics.

#	Sex	Age *	Diagnosis	Location	Tumor Size (cm) ^n^	Dose/Fractionation
1	M	81	urothelial carcinoma	LUL	3.6	50 Gy in 4 fractions
2	F	72	NSCLC (stage IA)	LLL	1.6	54 Gy in 3 fractions
3	M	69	colorectal adenocarcinoma	RUL	1.1	51 Gy in 3 fractions
4	M	49	colorectal adenocarcinoma	LLL	0.8	54 Gy in 3 fractions
5	F	64	colorectal adenocarcinoma	RLL	0.9	55 Gy in 5 fractions
6	M	62	NSCLC (stage IV), progressive nodule	RUL	2.5	50 Gy in 4 fractions
7	F	82	colorectal adenocarcinoma	LLL; RUL	1.8; 3.5	50 Gy in 4 fractions (both)
8	F	78	NSCLC (stage IV), progressive nodule	RLL	1.6	45 Gy in 3 fractions
9	F	71	colorectal adenocarcinoma	RLL	1.8	51 Gy in 3 fractions
10	M	63	NSCLC (stage IA)	RUL	1.4	54 Gy in 3 fractions
11	F	51	colorectal adenocarcinoma	LLL	3.2	55 Gy in 5 fractions
12	M	73	NSCLC (stage IB)	RLL	3.3	48 Gy in 4 fractions
13	M	76	NSCLC (stage IA)	RUL	2.1	50 Gy in 5 fractions
14	F	72	colorectal adenocarcinoma	LLL	1.1	54 Gy in 3 fractions
15	M	76	NSCLC (stage IA)	RLL	1.3	50 Gy in 4 fractions

* = age at tx initiation

^n^ = based on most recent CT or PET/CT prior to tx

### Fiducial marker placement

**Fig 1:**
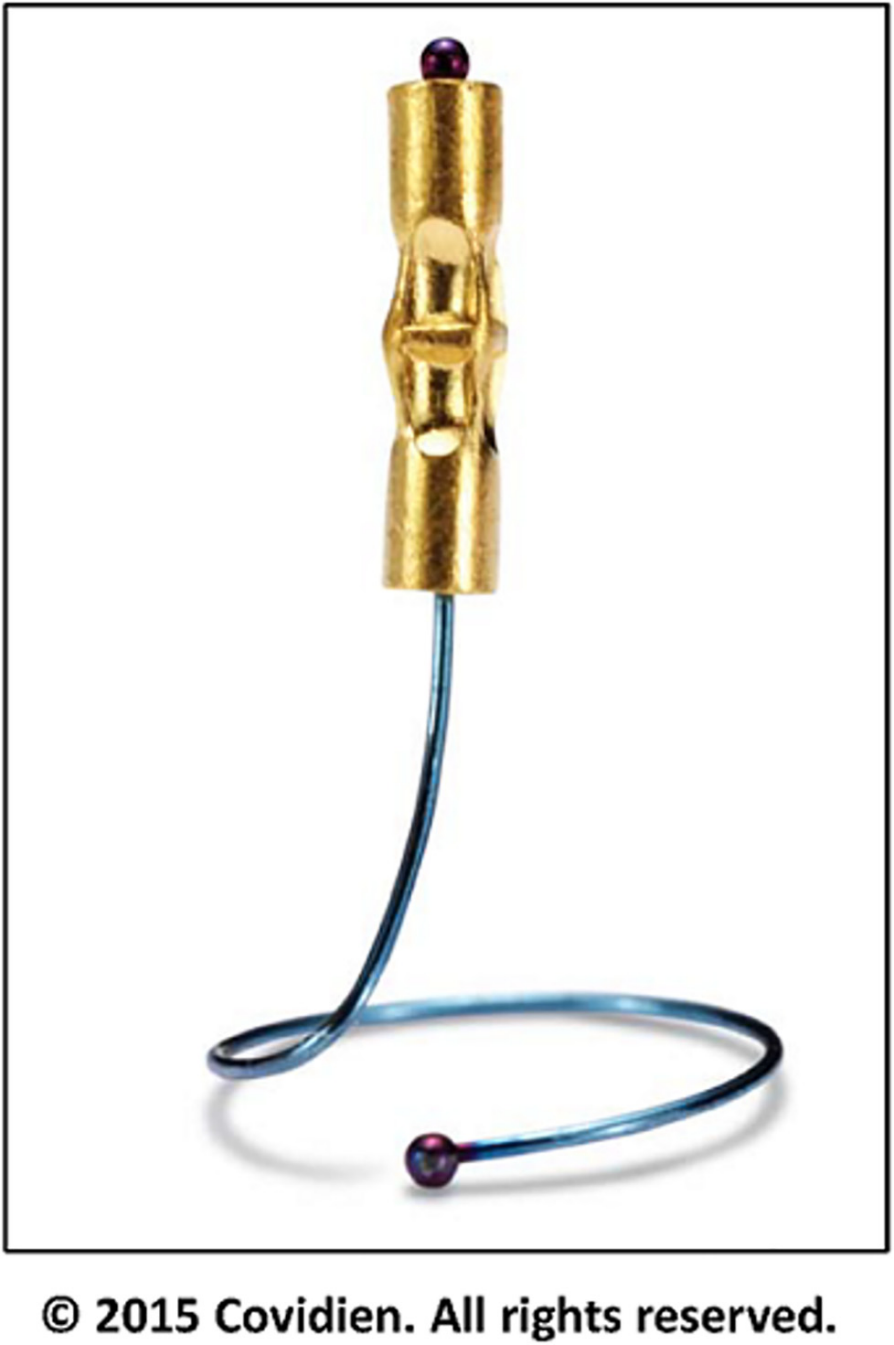
The SuperLock nitinol coil fiducial marker.

**Fig 2:**
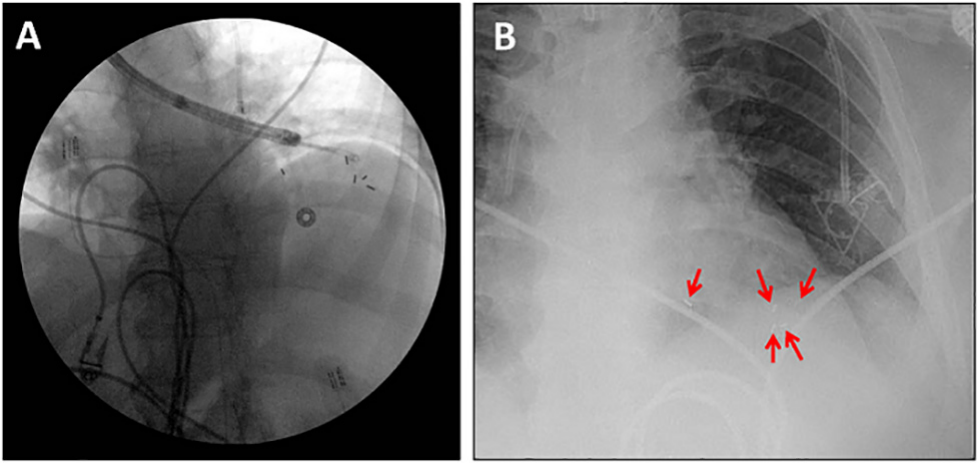
SuperLock nitinol coil fiducials deployed into the left lower lobe of the lung through electromagnetic navigation bronchoscopy. Five fiducials were deployed, four in or within 1–2 cm of the tumor, and one further away as visualized during the placement procedure (panel A), and at a post-procedure chest radiograph (panel B). The fiducials were centered around the tumor, which is not well-visualized in the kV images.

The SuperLock nitinol coil fiducial markers were used for all exhale breath-hold gated lung SBRT treatments. As shown in Fig 1, these fiducials are made of a 0.8 mm x 3.5 mm textured gold seed attached to a coiled nitinol wire, for an overall length of 7 mm. The seed was designed to minimize migration in the lung parenchyma, and appeared as radiopaque seeds on fluoroscopic imaging. Patients were identified at the time of physician consultation to see if they were appropriate candidates for the breath-hold gated SBRT protocol. All patients who consented to receive this treatment were referred to pulmonary or thoracic surgery for placement of SuperLock nitinol coil fiducials (n = 3 or 4) adjacent to their lung lesions (typically within 2 cm). The fiducial placement was performed through a minimally invasive electromagnetic navigated bronchoscopy procedure using the superDimension Navigation System (Covidien, Inc., Minneapolis, MN, USA). A mobile fluoroscopic C-Arm system (GE Healthcare, Waukesha, WI, USA) was used to monitor and confirm the fiducial placement, as shown in Fig 2. Patients were CT simulated no sooner than 40–48 hours after fiducial implantation.

### CT simulation and treatment planning

Our CT simulation protocol for lung SBRT breath-hold gated treatment included two steps: A 4D-CT scan followed by a standard planning CT scan with or without intravenous contrast. The 4D-CT scan was performed with abdominal compression (CIVCO Medical Solutions, Coralville, Iowa, USA) to determine the maximum tumor motion, which was then used to determine the type of planning CT scan (a free-breathing scan if the motion was ≤ 1 cm, or a breath-hold scan otherwise). If the tumor motion was larger than 1 cm, Respiratory Position Management (RPM, Varian Medical Systems, Palo Alto, CA, USA) was used to record the breath-hold level. Accordingly, patients were treated with a breath-hold gated SBRT technique. For this treatment, the target contour was the gross tumor volume (GTV) on the breath-hold CT, with recommended minimum 5 mm axial and 5–8 mm superior-inferior margins to generate the planning target volume (PTV). The variation in the superior-inferior margin depended on the tumor motion magnitude, 4D CT quality, and physician preference. A variety of treatment planning techniques, including 3D conformal treatment, intensity modulated radiotherapy (IMRT), or dynamic conformal arc therapy, were considered for the optimal dosimetric conformity, rapid dose fall-off, as well as faster delivery time. Treatments were delivery through a Varian TrueBeam Linac (Varian Medical Systems, Palo Alto, CA, USA), with the 6X flattening-filter-free beam and 1000 MU/min dose rate.

### Treatment procedure and imaging protocol

**Fig 3:**
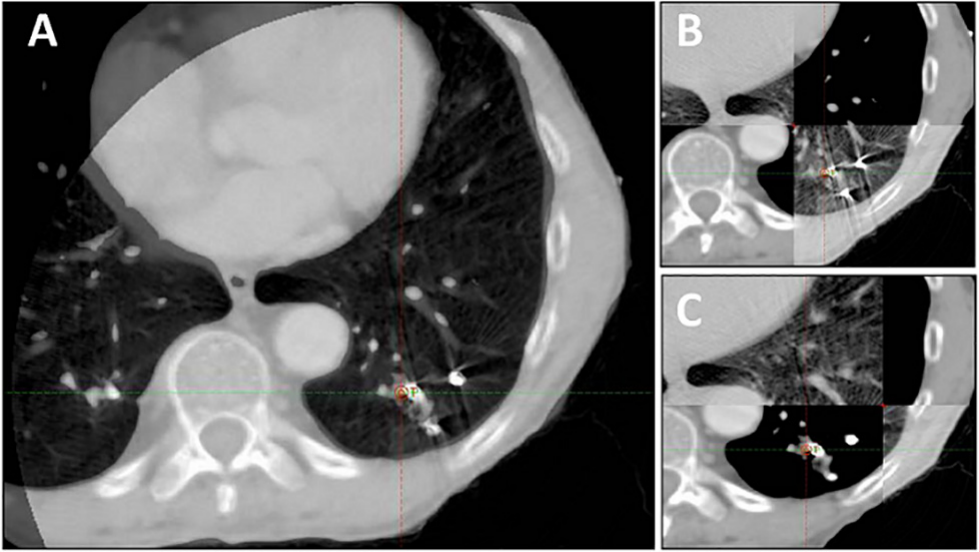
Breath-hold CT and CBCT (spotlight) matching with three fiducial markers in the left lower lobe of the lung, just prior to SBRT treatment. Panel A shows CBCT completely overlaid onto the planning CT images. Panel B-C shows the two-way sliding window matching between CBCT and planning CT images.

**Fig 4:**
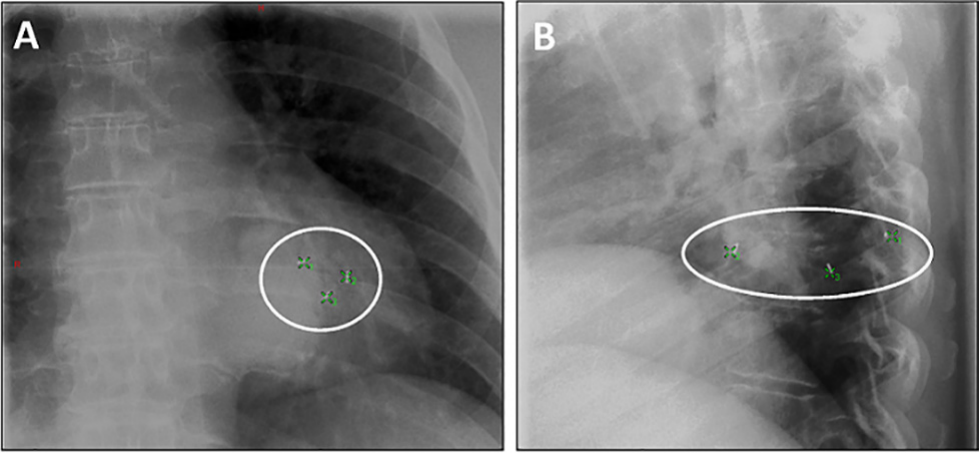
Position of three SuperLock nitinol coil fiducials used in two dimensional (2D) marker matching just prior to SBRT treatment for a left lower lobe lung tumor as visualized using on-board kV imaging (panel A: anterior-posterior image; panel B: lateral image). Three green crosses showed locations of the fiducials identified from the planning CT. The background images were 2D kV-kV pair images acquired prior to treatment.

Median interval between the planning CT and the first CBCT was 2 weeks. Subsequent CBCTs were taken at 1–2 day intervals, except during the weekend, with the goal of delivering 2–3 fractions per week. During each fraction of SBRT, the pre-treatment imaging sequence included at least one kilovoltage (kV) CBCT, which was acquired in the “Spotlight” mode with manual gating, as shown in Fig 3. Therapists started CBCT acquisition after the breath-hold trace settled in the gating window and stopped it before instructing patients to breath freely. It typically required 2–3 breath-holds to reconstruct a full Spotlight CBCT. In addition, 2D breath-hold kV-kV pair (2D-pair for short) images were used for bony anatomy alignment and 2D marker matching prior to or after the CBCT, as shown in Fig 4. Both fiducial registration and bony anatomy registration were considered and the final shift would take into account both and split the difference if they were not consistent. Within our study group, 11 out of 15 patients were imaged with a 2D-pair followed by a breath-hold CBCT. After the initial 2D-pair alignment, each patient was further aligned with the registration between the breath-hold CBCT and the planning CT. This latter alignment served as the primary registration and provided the final shifts prior to treatment. Patients 3 and 5 on the list were imaged with a breath-hold CBCT first followed by a 2D-pair, and patients 4 and 10 were imaged with a breath-hold CBCT only. This procedural variation depended solely on the attending physician’s preference. The CBCT scans were rigidly registered to the planning CT by minimizing the differences in fiducial locations, bony anatomy and target positioning in order to achieve the optimal fusion. Even with RPM monitoring, the breath-hold level can still vary significantly. Therefore, we typically performed a second series of breath hold gated imaging if the shifts were considered too large (greater than 3 mm), in order to confirm the shifts. Table positions were re-acquired after the first fraction imaging.

### Data acquisition and analysis

There were a total of 55 fiducials and 16 tumor targets. For each fraction, we compared the individual and group migrations of the fiducials between the post-implantation planning CT and the acquired CBCT for each fraction. The individual migration was defined as the averaged relative distance of each individual fiducial between the planning CT and the subsequent CBCTs. In other words, the migration of each fiducial was calculated based on the 3D coordinates on CBCTs with respect to its location in the planning CT. Therefore, the accuracy of CT-CBCT registration is essential for the individual migrations. The group migration was defined as the distance between the centroids of the fiducial group and the GTV. GTVs were contoured on the planning CTs and CBCTs in order to locate the geometric centers of the target. The group migration of each set of 3–4 fiducials was calculated based on the difference in fiducial-to-GTV centroid distances between the planning CT and the subsequent CBCTs. The fiducial-to-GTV centroid distance was calculated based on the 3D coordinates of the fiducial group centroid with respect to the GTV center on each CBCT.

## Results

**Fig 5:**
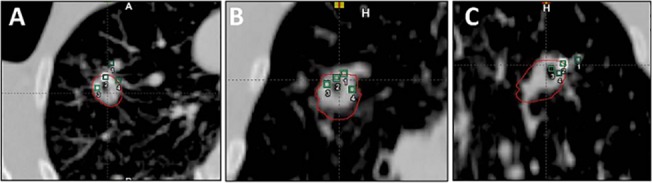
CBCT images of 4 fiducials placed in or near a tumor in the right upper lobe treated with SBRT in (A) axial, (B) frontal, and (C) sagittal views. The four green squares indicated the four fiducials identified on the planning CT.

**Fig 6:**
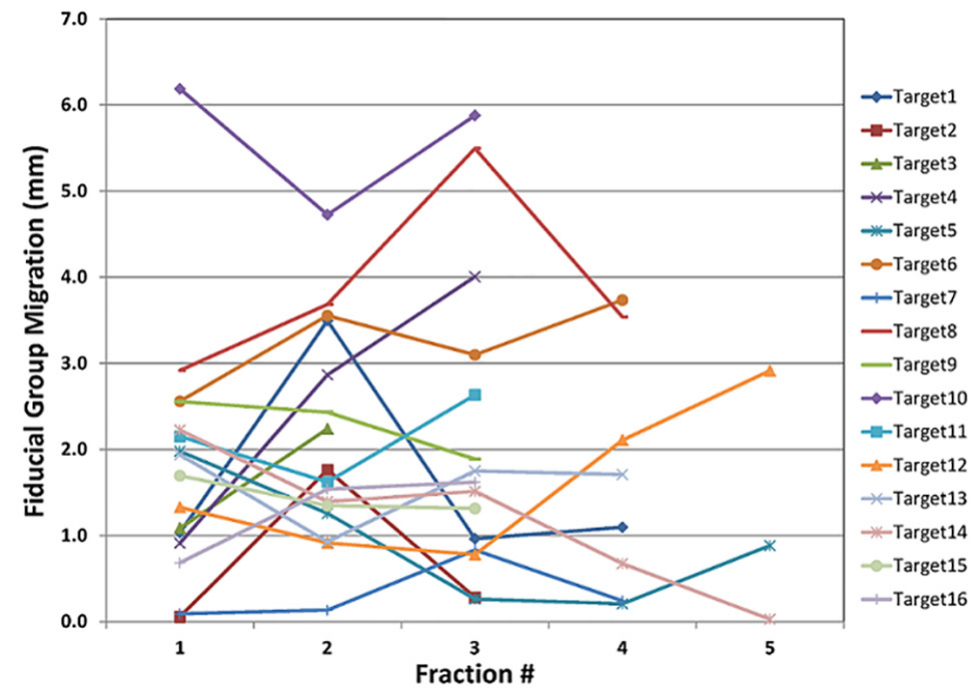
Fiducial group migration with respect to the GTV for all 16 targets.

**Fig 7:**
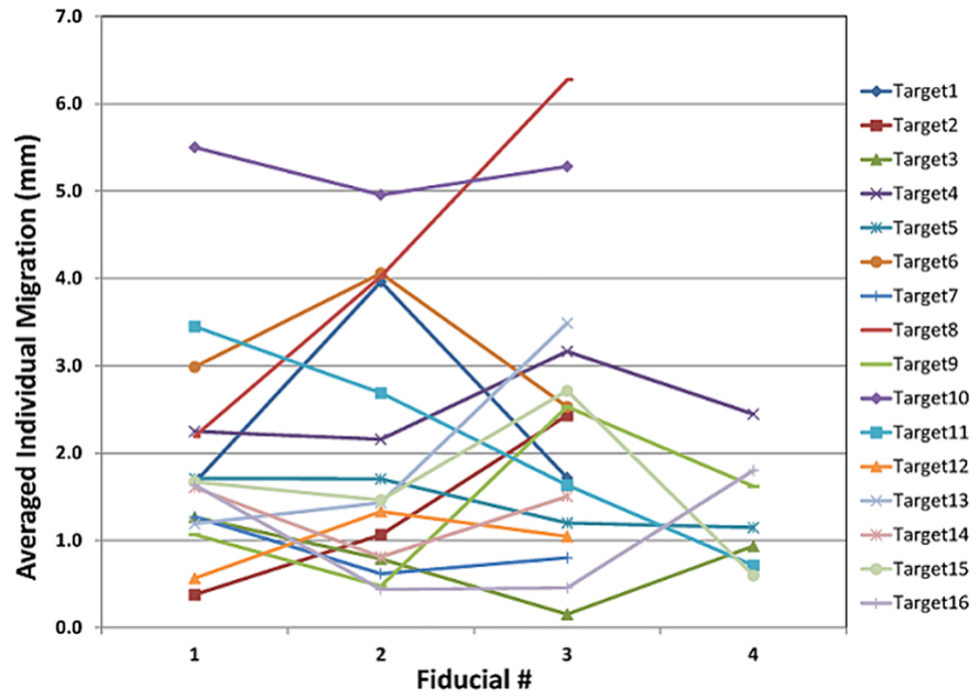
Fiducial averaged individual migration with respect to the planning CT for all 16 targets.

**Fig 8:**
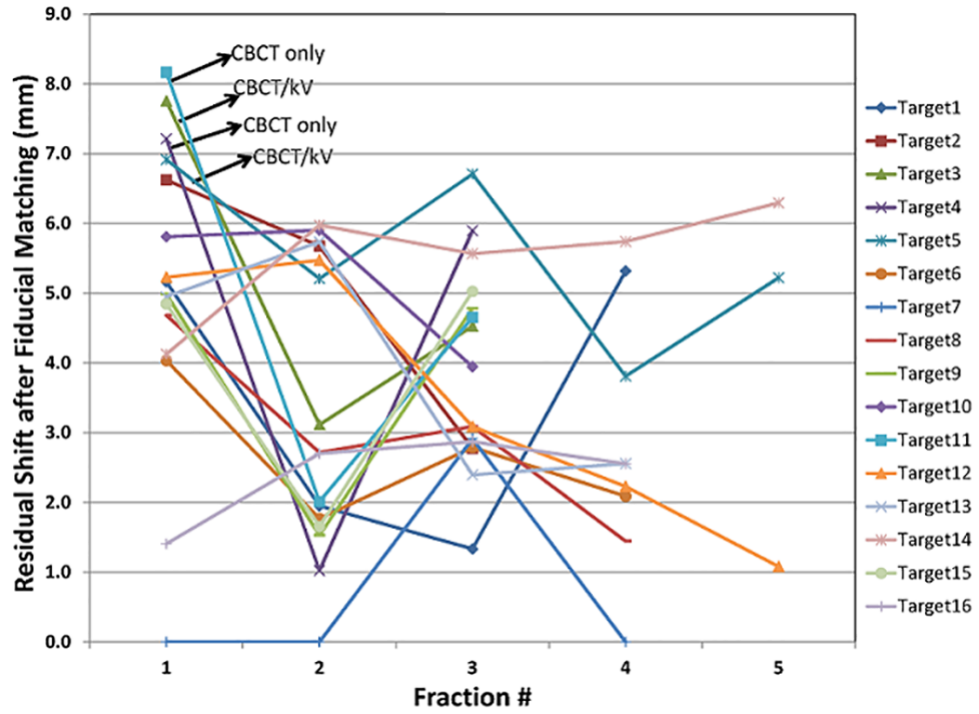
Residual shifts after the first imaging prior to the SBRT treatment for all 16 targets.

For the 15 patients undergoing SBRT in this study, the median number of SBRT fractions was 3, and the median number of fiducials placed in or near the tumor was 3. All fiducials implanted were readily visible on planning CT imaging, as well as the kV and CBCT images acquired on the day of treatment, reflecting a 100% retention rate. To enable the marker matching function on the Varian TrueBeam system, fiducials needed to be identified on the treatment planning system, as shown in Fig 5. As shown on Figs 3 and 5, there were low-degree streaky artifacts introduced by the fiducials on the CBCT images. Despite these artifacts, we found that the optimal visualization of the target on the CBCT was better when the fiducials were near the target, but not directly residing in it. For each fraction, we compared individual and group migrations of the fiducials between the planning CT and CBCTs. Between fractions, as shown in Table 2, the mean individual migration was 1.87 mm (range, 0.63–5.25 mm) with a standard deviation of 1.26 mm. The mean group migration was 1.94 mm (range, 0.03–6.19 mm) with a standard deviation of 1.45 mm. Overall, the majority of the fiducial markers had relative location changes of less than 5 mm. Figs 6 and 7 depict the group and averaged individual migrations for each target, respectively. Fig 6 shows the fiducial group migration for each fraction by target lesion, while Fig 7 shows average individual migration across all the fractions for each fiducial placed around a target lesion. Except for two patients (Target 8 and 10, Figs 6 and 7), the majority of the observed migrations were below 4 mm. Taking into account the measurement uncertainty inherent with the CBCT image quality and object smearing out with respiratory motion, we concluded this amount of migration is within our accepted tolerance based on the minimum PTV expansion of 5 mm. Fig 8 plots the shifts after the first imaging sequence (residual shifts) in the vector of all three directions across fractions for each target. As we mentioned previously, the majority of patients (11 out of 15) were imaged with 2D-pair followed by breath-hold CBCT. The shift adjustments made from 2D-pair were based on bony anatomy alignment and marker-marker matching in or around the target. As shown in Fig 8, the residual shifts indicated from the breath-hold CBCT were mostly within 6 mm (the vector of all three directions), after adjusting the patient’s positioning based on a 2D-pair for those 11 patients. The remaining patients (4/15) were imaged either with the breath-hold CBCT first followed by 2D kV pair (2), or with the breath-hold CBCT only (2). These four patients with the different imaging sequence or lack of 2D-pair were the ones with the largest shifts on the first fraction as indicated in Fig 8. Despite this, there is likely no clinical impact with these large shifts for these four patients, since the shifts were applied subsequently to correct for any setup misalignments. However, an additional gated imaging may be necessary for these patients in order to confirm the reproducibility of the imaging position.

**Table 2:** Maximum, mean, and standard deviation for fiducial individual and group migration.

Total Fiducials	55
Total Targets	16
Total Patients	15
**Individual Migration (mm)**	
Max	5.25
Min	0.63
Mean	1.87
Stdev	1.26
**Group Migration (mm)**	
Max	6.19
Min	0.03
Mean	1.94
Stdev	1.45

## Discussion

Pulmonary tumor motion is a significant problem both during and between SBRT treatments. Fiducials serve as one way to mitigate the effects of motion by allowing for improved IGRT. In this study, we have examined the migration of the grouped and individual intrapulmonary SuperLock nitinol coil fiducials centered around the SBRT target, in order to evaluate marker stability within the pulmonary parenchyma. Given the less dense nature of pulmonary tissue (typically ~1/3^rd^ dense compared with other soft tissues that commonly use fiducial markers for SBRT including liver and prostate), there is a real concern that fiducial markers migrate in lung tissue more than other organ sites. To our knowledge, this is the first study assessing potential marker migration of the SuperLock nitinol coil fiducials, which have been designed specifically for lung tissue.

Overall, we observed the majority of fiducial migrations to be less than 5 mm. The mean migrations for individual and group markers were both under 2 mm, and the standard deviations for the individual and group markers were under 2 mm as well. As fraction number increased, we did not find substantial or consistent changes in the group inter-marker migrations (Fig 6), suggesting that radiation did not result in significant organ-tissue deformation during the course of treatment. These results may have implications for correctly targeting lung tumors using these implanted fiducial markers. This study is a single center retrospective study with 15 patients/16 tumors included, and the authors realize that this may not be necessarily representative of a much larger population of patients. However, the use of the three fiducial combination may enhance the accuracy of treatment over single fiducial alignment, by avoiding issues of organ deformation (which we did not detect in this study). Indeed, other studies have demonstrated that more than a single fiducial improves target localization [22].

Prior to the introduction of this new SuperLock nitinol coil fiducial marker, commonly used fiducial markers for pulmonary tumors include “seed” and “coil” markers [20,21,23–27]. These commercially-available markers have various shapes (sphere [20,25] or cylindrical [21,23,24,26,27]) and sizes (1.5 mm–2 cm). In terms of the retention rate, the present study reports a 100% rate for these nitinol coil markers, which is equivalent or superior to other marker shapes (close to 100% for the coil markers [23,24] and 85–95% for the sphere marker [23–25]). In terms of the rate and extent of marker migration, previous studies have observed a rate of 12% of markers experiencing large migrations (>5 mm) for both seed and coil markers [21,23,25]. The present study noted a 7% rate of the fiducial markers having a >5 mm migration. A follow-up study with a larger population of patients is necessary to further establish the stability of these fiducial markers.

As shown in Fig 8, alignment based on the three fiducial’s group geometric center should provide a very reasonable alignment of the lung tumor within the radiation field/isocenter, which was confirmed by the residual shifts observed from the breath-hold CBCT-to-CT soft tissue matching just prior to treatment. This finding indicates that in the absence of CT-CBCT matching, the intrapulmonary implanted fiducials should still allow for a reliable and simple localization approach for treatment of the lung tumor. However, if both 2D-pair and CBCT imaging techniques are available, the sequence of 2D-pair followed by CBCT is recommended based on our findings. This imaging sequence largely decreases the residual shifts after the first imaging, which minimizes the variations inherent in a breath-hold imaging acquisition and provides improved confidence in patient positioning. In our study, the four patients who received CBCT first or CBCT only had the largest residual shifts, which indicated that an additional imaging may be necessary in order to confirm the reproducibility of the imaging position. This could subsequently increase patient’s discomfort by requiring additional breath-holds, thereby prolonging total treatment time. Respiratory-gated treatments can also be facilitated by monitoring the fiducial motion during the breathing cycle, using MV or kV fluoroscopic or cine-mode imaging prior to and/or during treatment. Given that re-irradiation is being considered more frequently in the setting of recurrent lung cancer [28–30], fiducial based SBRT approaches may become more common, which allow for decreased volumes of normal lung irradiated and decreased overlap from prior treatment fields.

Fiducial placement via CT guidance is typically associated with a high rate (from 13% [5] to over 60% [31–33]) of iatrogenic pneumothorax. All of the fiducials in this study were placed by electromagnetic navigation bronchoscopy (ENB) [34,35]. While fiducials are likely to aid with target localization and enhance accurate treatment delivery, the implantation of fiducials is not without potential risks of toxicity. In addition to the implantation procedure carrying its own set of risks, a number of case reports have also been published describing dislodgement or migration of fiducials, sometimes with significant clinical consequences [36–38]. Thus, patients need to be aware of these risks associated with the implantation and use of fiducials, and physicians need to weigh these risks compared with the benefits of improved accuracy with these markers for SBRT. One potential way to avoid additional risks and minimize more procedures are to have fiducials implanted at the time of a diagnostic biopsy of a pulmonary nodule or mass, particularly if the physician performing the procedure deems the patient a potential candidate for SBRT (e.g. medically inoperable).

In summary, we have demonstrated that the SuperLock nitinol coil fiducial markers demonstrated minimal migration or displacement from the time of treatment planning through treatment delivery in patients receiving lung SBRT. These fiducials also retained their relative position to the tumor throughout the course of SBRT. Fiducial implantation helps to improve the efficiency and accuracy of patient positioning through 2D-pair imaging and marker matching techniques. To our knowledge, this is the first report characterizing migration of these particular fiducials in lung tissue. Thus, SuperLock nitinol coil fiducials are reliable surrogates in cases where the target is not clearly visualized on the planning CT and/or CBCT, and will serve as appropriate markers for real-time tracking or gated SBRT techniques to enhance treatment delivery.

## Supporting Information

S1 File Raw data for Figs 6 and 7. (XLSX)

S2 File Raw data for Fig 8. (XLSX)
